# Assessing the Economic Efficiency of Physician On-call Payments

**DOI:** 10.7759/cureus.3768

**Published:** 2018-12-24

**Authors:** Mahesh B Shenai, Barton L Guthrie, Leon Moores

**Affiliations:** 1 Neurosurgery, Inova Fairfax Hospital, Fairfax, USA; 2 Neurosurgery, University of Alabama at Birmingham, Birmingham, USA

**Keywords:** relative value unit, on call, fair market value, compensation, subspecialty call

## Abstract

On-call services provided by physicians are critical to the function of a robust healthcare delivery system, but such services are not generally accounted for by standard physician productivity metrics, such as the work relative value unit (wRVU). There is significant diversity on how physicians are compensated, if at all, for these on-call services. Simultaneously, there exists a considerable shortage, particularly in the surgical subspecialties, for on-call coverage – most commonly in rural and underserved communities. While we agree that “call” services should undergo standardized valuation, we suggest that the wRVU is an ill-posed metric for this purpose as its primary role is to value discrete physician services provided to patients. In contradistinction, “call” is a physician service to a hospital – the disproportionate beneficiary of the service. We maintain that systemic and regulatory factors undervalue physician on-call compensation relative to the hospital’s value chain and lead to call shortages that impact patient care and foster inequity. Finally, we urge subspecialty professional organizations to develop guidelines for call valuation.

## Editorial

Physician on-call services are a fundamental need to hospitals and healthcare delivery networks, in order to provide uninterrupted urgent and emergent care. Nevertheless, a large fraction of the roughly 5200 emergency rooms in the United States cannot procure full coverage for subspecialists, particularly subspecialists such as neurosurgery, cardiothoracic surgery, and orthopedic surgery. Consequently, uncovered facilities depend on time-consuming and costly transfers to higher-level centers that provide these advanced services. While historically many physicians viewed call as a “duty” and “privilege”, many now view it as a transactional requirement [1] that should be fairly compensated. However, with hospitals’ increasing focus on financial performance the fair valuation of physicians’ call services has generally (and predictably) lagged its true financial value.

While call can yield variable levels of productivity through Evaluation and Management (E&M) and procedural CPT codes, a physician on call provides a greater service than those work relative value units (wRVUs). In addition, there are professional and personal costs that should be considered. The volatile nature of call can disrupt outpatient clinic or elective cases, causing patient defections or negatively impacting patient satisfaction. Personally, the stress and unpredictability of emergency patient care can detract from family, leisure and non-clinical pursuits – increasing the risk for physician burnout. This difference could be accounted for by a “non-CPT” stream of compensation, imputed in an additive and transparent manner.

As an attempt to compensate physicians for the effort and time, a stipend is sometimes remitted by a cash payment or guaranteed subsidy for uninsured care. For hospital-employed physicians, the value of call may be (albeit ambiguously) imputed into the physician’s base salary. These methods confirm that the act of being “on call” has independent value – with varying inputs such as subspecialty, geography, presence of assistants, risk, or call frequency. According to the Medical Group Management Association (MGMA) Medical Directorship and On-Call Compensation Report, median rates of general emergency department (ED) call can range from $500 to 1,000/day, and between $1,500 and $2,300 for subspecialties involved in trauma care. Based on the wRVU conversion factor (which differs institutionally, and by subspecialty), this could range between 10 and 45 wRVUs/call, as suggested for cardiothoracic surgery [1].

Do these ranges represent the value of on-call services? We maintain that reported median compensation for these services is significantly undervalued, as evidenced by the locum tenens industry’s attempts to fill open assignments with premium compensation. The supply/demand mismatch this level of compensation demonstrates has a real human impact, as the lack of on-call subspecialists in rural and underserved areas delays emergency procedures, leading to mortality and morbidity. Lack of subspecialty call in remote hospitals also leads to costly, and often unnecessary transfers to tertiary hospitals [2]. Society has a stake in solving this market gap.

Stark laws and the Antikickback Statute (AKS) impact the efficiency of this market. In 2007 and again in 2012, the Office of the Inspector General (OIG) rendered advisory opinions on the subject [3], opining that arrangements be “commercially reasonable” and within “fair market value” (FMV). Intended to limit hospital inducement of referrals by physicians, these statements negatively bias the valuation of call services, due to the systems’ perceived regulatory risk. FMV is often based on survey data from MGMA and consultant agencies, which report prior year metrics and strongly influence current FMV rates, suppressing market-driven escalation for on-call FMV. Both of these regulatory and surveying phenomena contribute to the disparity between realized and economically-efficient valuation.

Specialty physician call services impact on the entire hospital’s value chain from DRG-related payments for emergency room visits, admissions, laboratory/radiology studies, and procedures related to the subspecialists oversight. Vallier et al. [4] found hospitals generate a net revenue of 7.81 times the professional revenue earned by an orthopedic trauma surgeon. Based on available data, a neurosurgeon may directly generate $300 per wRVU [5] for the hospital, while collecting only $60-100/wRVU himself.

Subspecialist call can impact hospital designations and accreditations (trauma level or centers of excellence) that are extremely lucrative to a hospital’s bottom line via both patient volume and federal and research funding. The Case Mix Index (CMI), a CMS measure of the average acuity of admitted patients, impacts overall reimbursement. Because of this value hospitals may be willing to pay specialists for call but are limited by regulatory restrictions described above. In the current market a hospital would pay $4500/day to a locum tenens agency, to staff neurosurgical call, more than 2.5 times the realized median daily on-call compensation for neurosurgery in 2017 ($1680/day) [5]. Regulatory risk is minimal with locum physicians since a referral relationship does not exist and thus is a better representation of a hospital’s measure of on-call FMV. FMV depends on numerous market factors, but this wide variation indicates the FMV is financially undervalued – not to mention the potential societal benefit of prompt and local subspecialist care.

To this end, we submit that the accurate fair market valuation of these services could allow supply and demand to better converge. Until a comprehensive discussion occurs regarding fair valuation for on-call services dangerous on-call shortages, particularly in rural and underserved areas, will continue to exist. We encourage professional subspecialty organizations to undertake such analyses and formulate guidelines for fair but accurate valuation of on-call services.
